# Pills or Push-Ups? Effectiveness and Public Perception of Pharmacological and Non-Pharmacological Cognitive Enhancement

**DOI:** 10.3389/fpsyg.2015.01852

**Published:** 2015-12-02

**Authors:** Lucius Caviola, Nadira S. Faber

**Affiliations:** ^1^Department of Experimental Psychology, University of OxfordOxford, UK; ^2^Oxford Martin School, University of OxfordOxford, UK

**Keywords:** cognitive enhancement, neuroenhancement, methylphenidate, modafinil, caffeine, physical exercise, computer training, sleep

## Abstract

We review work on the effectiveness of different forms of cognitive enhancement, both pharmacological and non-pharmacological. We consider caffeine, methylphenidate, and modafinil for pharmacological cognitive enhancement (PCE) and computer training, physical exercise, and sleep for non-pharmacological cognitive enhancement (NPCE). We find that all of the techniques described can produce significant beneficial effects on cognitive performance. However, effect sizes are moderate, and consistently dependent on individual and situational factors as well as the cognitive domain in question. Although meta-analyses allowing a quantitative comparison of effectiveness across techniques are lacking to date, we can conclude that PCE is not more effective than NPCE. We discuss the physiological reasons for this limited effectiveness. We then propose that even though their actual effectiveness seems similar, in the general public PCE is perceived as fundamentally different from NPCE, in terms of effectiveness, but also in terms of acceptability. We illustrate the potential consequences such a misperception of PCE can have.

## Introduction

Cognitive enhancement is defined as “interventions in humans that aim to improve mental functioning beyond what is necessary to sustain or restore good health” (Dresler et al., 2013, p. 529). There are several means for such cognitive enhancement, both pharmacological (PCE) and non-pharmacological (NPCE). We summarize literature on the effectiveness^1^ of six often discussed and prevalent potential enhancements, namely caffeine, methylphenidate, and modafinil for PCE, and computer training, physical exercise, and sleep for NPCE. We conclude that PCE is not more effective than NPCE and discuss the physiological reasons for this limited effectiveness. We then illustrate that although they have similar effect sizes, PCE is perceived by the general public as fundamentally different from NPCE, in terms of effectiveness and but also acceptability.

## Effectiveness of pharmacological cognitive enhancement

### Methylphenidate

Methylphenidate is a pharmacological psychostimulant of the phenethylamine and piperidine classes and best known under its marketing label Ritalin®. It acts as a reuptake inhibitor, increasing dopamine and norepinephrine levels (Sulzer et al., 2005). Although methylphenidate is usually prescribed for attention deficit hyperactivity disorder, evidence suggests that it can enhance cognitive performance in healthy individuals.

In meta-analyses, it was found that methylphenidate exhibits large positive effects (*d* = 1.4; Repantis et al., 2010) on memory performance, that delayed episodic memory is improved by a moderate (*d* = 0.45; Ilieva et al., 2015), and short-term episodic memory by a smaller effect size (*d* = 0.20; Ilieva et al., 2015). This suggests that methylphenidate primarily enhances memory consolidation but neither encoding nor retrieval (cf. McGaugh and Roozendaal, 2009). A review concluded that verbal learning appears to be improved by methylphenidate, whereas visual learning remains unaffected (Linssen et al., 2014). Methylphenidate further improves working memory. Small, but robust, positive effects on spatial working memory have been reported in many studies (for reviews, see Repantis et al., 2010; Franke et al., 2014; Linssen et al., 2014; Ilieva et al., 2015). Further, methylphenidate has been shown to improve inhibitory control and speed of processing (Ilieva et al., 2015). The effects of methylphenidate on attention are less consistent. Most studies have reported no significant improvements in attention (cf. Repantis et al., 2010), or even negative effects (e.g., Rogers et al., 1999). However, a few studies have found small improvements in attention and vigilance (cf. Linssen et al., 2014). It has been speculated that methylphenidate might also affect motivational and emotional functions. However, although some data seem to support this hypothesis (Volkow et al., 2014), to date there is too little evidence to draw definite conclusions.

The enhancing effects of methylphenidate are greater in low-performing than high-performing individuals (Finke et al., 2010). Methylphenidate can even impair the performance of high-performers (Mattay et al., 2000; de Wit et al., 2002; Farah et al., 2009). One study, for example, has shown that methylphenidate can disrupt attentional control in certain individuals (Rogers et al., 1999). Further, methylphenidate consistently increases heart rate, and increased blood pressure, headache, anxiety, nervousness, dizziness, drowsiness, and insomnia have been reported occasionally (Repantis et al., 2010).

### Modafinil

Modafinil is a wakefulness-promoting agent originally developed to treat narcolepsy, but it is also applied as PCE (Sahakian and Morein-Zamir, 2007). The neuropsychology of modafinil is not yet well understood. It is assumed that dopamine and norepinephrine are involved in its mechanisms (Ballon and Feifel, 2006; Volkow et al., 2009).

Modafinil consistently improves attention in non-sleep deprived as well as sleep-deprived healthy individuals (for reviews, see Repantis et al., 2010; Franke et al., 2014; Battleday and Brem, 2015). In particular, experiments have shown improvements in sustained attention (Baranski et al., 2004; Randall et al., 2005; Dean et al., 2011) and selective attention (Schmaal et al., 2013). The effects of modafinil on memory are less clear. Some studies report beneficial effects of modafinil on spatial and numeric working memory (Müller et al., 2004). However, a review of 31 randomized controlled studies reported no significant changes in memory (Repantis et al., 2010).

It is assumed that the effects of modafinil strongly depend on the individual baseline performance (Randall et al., 2005). Similar to methylphenidate, modafinil appears to positively affect low-performing individuals to a greater extent than high-performing individuals (Finke et al., 2010). Further, the effects of modafinil are strongest for cognitively demanding tasks (Müller et al., 2013). However, it potentially impairs creative and flexible thinking (Müller et al., 2013; Mohamed, 2014) and can increase feelings of overconfidence in judgment (Baranski et al., 2004). Further, Repantis et al. (2010) reported that potential, but rare, side effects of modafinil are headache, dizziness, gastrointestinal complains, nervousness, restlessness, and insomnia.

### Caffeine

Caffeine is an adenosine receptor antagonist, applicable inter alia in the forms of coffee, tea, or energy drinks. It is assumed that caffeine stimulates neural activity through higher noradrenaline emission (Smith et al., 2003; Ferré, 2008).

Several studies have shown that caffeine improves sustained attention and alertness in simple tasks (for a review, see Einöther and Giesbrecht, 2013). The beneficial effects in complex tasks, however, are less consistent (Rogers and Dernoncourt, 1998; Heatherley et al., 2005). Further, caffeine can improve both encoding and response speed to new stimuli (Riedel et al., 1995; Warburton et al., 2001), as well as long-term memory consolidation (Borota et al., 2014). However, it is not clear whether reported memory improvements could be due to an increase in attention during encoding (Nehlig, 2010).

Effects of caffeine are moderated by level of habitual intake (Attwood et al., 2007), age (Nehlig, 2010), and even personality (Smith, 2002). Caffeine can have negative effects at high doses (from ~400 mg). Such high doses can reduce motivation (Lieberman, 1992), and potentially also cognitive performance. Hasenfratz and Bättig (1994), for example, reported that doses of 420 mg doses of caffeine resulted in more commission errors and slower processing rate in cognitive tasks than lower doses. Further, withdrawal of heavy caffeine consumption can result in adverse side effects including headaches, increased subjective stress, fatigue, and decreased alertness (e.g., Dews et al., 2002; Juliano and Griffiths, 2004).

In sum, evidence regarding methylphenidate, modafinil, and caffeine shows that PCE can significantly improve certain cognitive functions healthy individuals. Most effects, however, are only moderate in size. Further, they are moderated by different factors, prominently baseline performance, and PCE dose, and improvements in one domain seem to go along with impairments in another. In other words, none of the three reviewed PCEs appears to be able to radically enhance cognition. Why not?

## Explanations for the limited effectiveness of PCE

**Figure 1:**
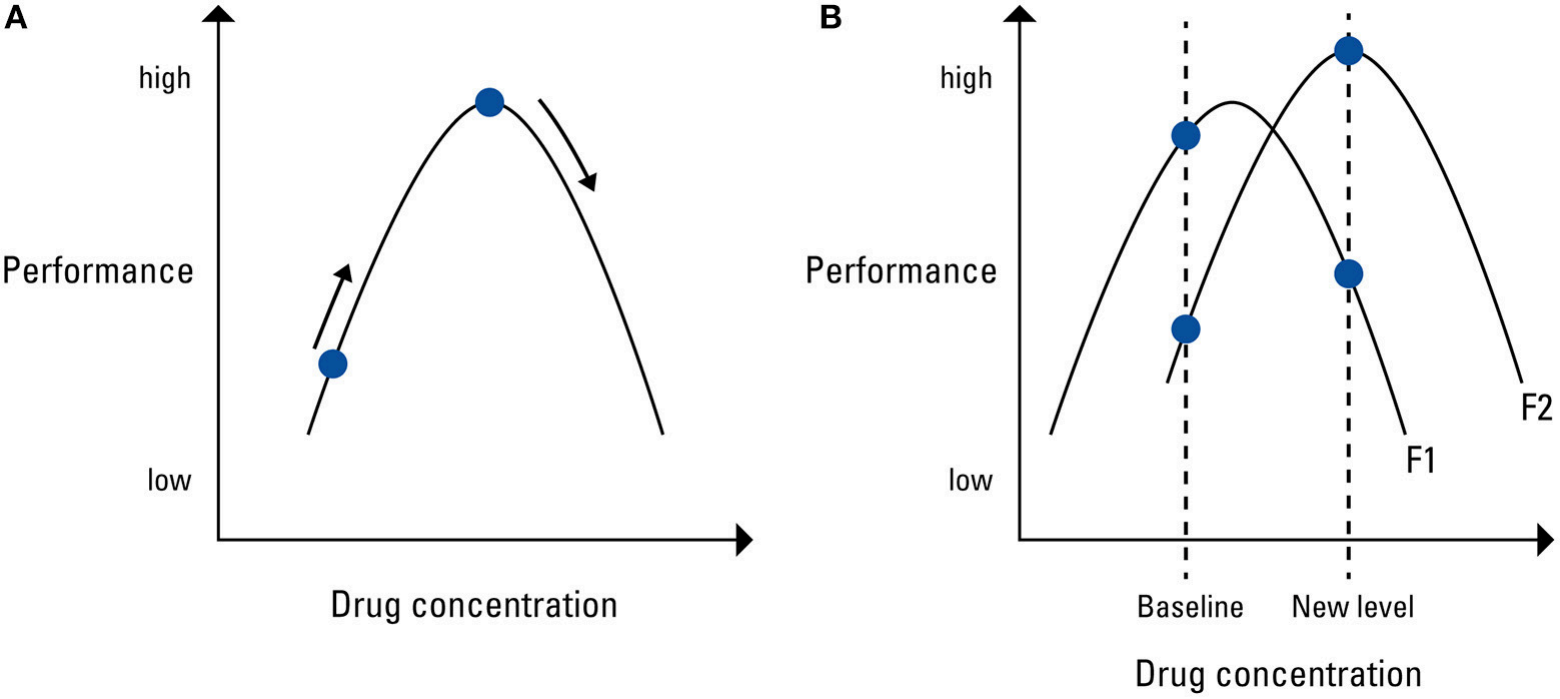
**(A)** Schematic display of the inverted-U shaped function between positive effect on cognitive performance and drug concentration in the brain. **(B)** An increase in substance level might improve one cognitive function but impair another. Adapted from Husain and Mehta (2011).

The pharmacological dynamics of PCE are not yet well understood. Many PCEs influence the levels of neuromodulators such as dopamine or serotonin, whose effects are complex and intertwined. PCE has been described to show an inverted U-shaped relationship between cognitive performance and dosage (Husain and Mehta, 2011; cf. Figure 1): evidence suggests that methylphenidate, modafinil, and caffeine are capable of enhancing certain cognitive functions up until a certain point, at which increased consumption will lead to cognitive decline. This is because both too high and too low concentrations of a certain neurotransmitter can impair cognitive function (Figure 1A; e.g., Hannestad et al., 2010). Accordingly, low baseline performers gain more benefits from PCEs than high baseline performers do, who might already exhibit optimal neurotransmitter concentration (Finke et al., 2010).

Further, improvements in one cognitive function often seem to be accompanied by impairments in another (Figure 1B). This is because an increase in substance level might improve one cognitive function (F1) but at the same time impair another (F2) due to differential drug sensitivity (Husain and Mehta, 2011).

## Effectiveness of non-pharmacological cognitive enhancement

### Computer training

Specifically designed computerized training programs can enhance cognitive functions (for a review, see Dresler et al., 2013). In particular, improvements in memory, attention (Smith et al., 2009), visual processing speed (Parsons et al., 2014), and executive functions (Basak et al., 2008; Nouchi et al., 2012) have been demonstrated, with effects lasting over a period of up to 3 months (Mahncke et al., 2006). A notable body of research has focused on the enhancement of working memory through computerized tasks with increasing difficulty over time. Such tasks can also improve executive functions and fluid intelligence (Jaeggi et al., 2008; Bergman Nutley et al., 2011), although the transferability to performance in every-day life has been questioned (Dahlin et al., 2008; Redick et al., 2013).

Commercial computer games can also improve cognition (Dresler et al., 2013). The evidence is particularly strong for the improvement of visual skills, including spatio-visual resolution (Green and Bavelier, 2007), mental rotation (Okagaki and Frensch, 1994), contrast sensitivity (Li et al., 2009), visual search (Castel et al., 2005), tracking of object color and identity (Sungur and Boduroglu, 2012), spatio-visual attention (Green and Bavelier, 2003), and the number of objects that can be attended (Achtman et al., 2008). Further, regular gamers appear to have improved cognitive flexibility (Colzato et al., 2010), multi-tasking ability (Strobach et al., 2012), enumeration skills (Green and Bavelier, 2006), and psychomotor skills (Kennedy et al., 2011).

The effect sizes of computerized training and games range from medium to large, depending on the trained and tested cognitive domain (Mahncke et al., 2006; Smith et al., 2009; Schmiedek et al., 2010). It is not clear, however, whether these effects can be explained by the similarity of the perceptual and attention tasks to the training programs (Oei and Patterson, 2014), and the extent to which they transfer to untrained tasks in real environments (Okagaki and Frensch, 1994; Fuyuno, 2007; Owen et al., 2010). There is no evidence of substantial negative side effects of computer training.

### Physical exercise

Acute exercise, in the form of brief bouts of exercise or high intensity training such as anaerobic running, can improve cognitive functions (for reviews, see Tomporowski, 2003; Lambourne and Tomporowski, 2010; Dresler et al., 2013; for methodological criticism, see Dietz, 2013). The cognitive enhancing effects of acute physical exercise have been linked to an increase in motivation and general arousal level (Brisswalter et al., 2002). Acute exercise may cause a similar physiological response as physical stress does, which has been linked to better episodic memory consolidation (Weinberg et al., 2014). Acute exercise improves memory performance by a medium effect size, but the effects vary depending on the specific type of exercise (Lambourne and Tomporowski, 2010). In particular, speed of learning (Winter et al., 2007), episodic memory (Weinberg et al., 2014), and general long-term memory (Coles and Tomporowski, 2008) can be improved. Some of these effects can persist over a period of up to 48 h after exercising (Weinberg et al., 2014).

Regular exercise has been shown to increase brain volume in gray and white matter regions (Colcombe et al., 2006). In particular, the size, cerebral blood flow, and connectivity of the anterior hippocampus, an area responsible for spatial memory, are increased through exercise (Burdette et al., 2010). Regular exercise can improve memory, attention, executive functions, and processing speed in general (Hillman et al., 2008; Smith et al., 2010). It also seems to improve academic performance, intelligence, perceptual, mathematical, and verbal skills in school-aged children (Sibley and Etnier, 2003).

Excessive acute exercise can lead to fatigue, dehydration, and decreased blood glucose levels, which can impair cognitive functions such as long-term memory (Cian et al., 2000, 2001; Grego et al., 2004). There is no evidence of negative side effects for regular exercise.

### Sleep

Sleep exhibits positive effects on cognition, particularly on memory (for a review, see Dresler et al., 2013). The underlying mechanisms are not yet understood. In particular, it is not clear whether the improved memory is due to active consolidation during sleep or to passive homeostatic mechanisms (Tononi and Cirelli, 2003). Studies suggest that neuronal patterns are reactivated during sleep, indicating a replay of memories (Wilson and McNaughton, 1994; Ji and Wilson, 2007; Diekelmann, 2014), and potentially promoting the formation of new neuronal connections (Yang et al., 2014).

Sleep can improve memory beyond the normal condition in rested/non-sleep deprived individuals (e.g., Jenkins and Dallenbach, 1924; Fischer et al., 2002; Diekelmann and Born, 2010), also memories acquired after sleep (Diekelmann, 2014). While the positive effects of sleep on declarative memory are moderate (Gais et al., 2006), the effects on procedural and perpetual memory can be very large (Karni et al., 1994; Fischer et al., 2002). Sleep can also increase creativity (Dresler, 2012) by triggering creative insights (Ritter et al., 2012) and speeding up problem solving (Wagner et al., 2004). Increased creativity has been particularly linked to REM sleep (Cartwright, 1972; Glaubman et al., 1978), the sleep stage in which most intense dreaming occurs.

Even naps (of 6 min or more) during the daytime can improve some memory systems to a similar degree as a whole night of sleep in non-sleep-deprived individuals (Mednick et al., 2003; Lahl et al., 2008).

Individual factors such as gender, hormonal level, and mental health moderate the effects of sleep (Genzel et al., 2009; Dresler et al., 2010). Further, there is evidence that too much sleep can impair cognition in the long run. A number of correlational studies have shown that sleep for more than 9 h per 24 h is associated with impaired cognitive function in elderly individuals (e.g., Benito-León et al., 2009; Devore et al., 2014). However, no causal connection has been demonstrated.

In sum, the reviewed evidence suggests that computer training, physical exercise, and sleep can moderately enhance cognitive functions. It appears, therefore, that NPCE techniques are similarly effective as current PCE techniques. Whether the effects of NPCE are limited by analogous restrictions with inverted-U shaped relationships like those for PCE is not yet clear.

## Public perception of pharmacological and non-pharmacological cognitive enhancement

The evidence presented above suggests that both currently available PCE (methylphenidate, modafinil, caffeine) and NPCE (computer training, physical exercise, sleep) are moderately effective in improving cognition. However, their effects are dependent on individual conditions, situational conditions, and the cognitive domain under study. There are a lack of experimental studies that directly compare the effects of PCE to NPCE, and it is difficult to undertake comparative meta-analyses of those studies that do exists, as they focus on different aspects of cognition (cf. Franke et al., 2014). Hence, we cannot draw a definite conclusion whether PCE or NPCE is more effective. Our qualitative analysis, however, suggests that the PCEs available to date are not more effective than NPCEs.

This, however, stands in sharp contrast to how people perceive PCE as opposed to NPCE. The general public views PCE—with the exception of caffeinated beverages—as fundamentally different from NPCE, both in terms of effectiveness and in acceptability. Several studies confirm this.

Most lay people would not even consider NPCE to be a form of cognitive enhancement. It is hard to imagine someone being concerned about their competitor going for a run to outperform them at the job interview. This is very different from the scenario where the competitor takes a “smart pill.” Lay people overestimate the effectiveness of PCE (Ilieva et al., 2013), and they express strong negative views toward its use (for a review, see Schelle et al., 2014). Unfairness is a particularly relevant concern in competitive settings (Faber et al., under review; also cf. Santoni de Sio et al., in press), at least when the explicit goal of PCE use is to improve cognition (Faber et al., 2015a). Such worries are unheard of in the case of NPCE, and they might in many cases be primarily rooted in the novelty or “unnaturalness” of PCE (Caviola et al., 2014), rather than justified threats to values like fairness posed by PCE.

Such a gap between the actual effectiveness of PCE as compared to NPCE should be seen as more than an interesting phenomenon of lay psychology, as real life phenomena could arise from it. There is a lively debate on the ethics of cognitive enhancement (for overviews, see Bostrom and Sandberg, 2009; Maslen et al., 2014), and scientists warn about overenthusiasm about the possibilities current PCEs offer (e.g., Farah, 2015; Sahakian et al., 2015). Such an overestimation of PCE effectiveness (paired with an underestimation of potential side-effects) could on the one hand lead to calls for certain people to take such substances, for example when they work in jobs with a high responsibility for other people's lives (for discussions see e.g., Santoni de Sio et al., 2014; Maslen et al., 2015). On the other hand, it could also lead to severe stigmatization of users in competitive settings (Faulmüller et al., 2013).

Moreover, the views individuals hold of PCE can alter how PCE influences performance, namely when these individuals act in groups (Faber et al., 2015b). Whether or not PCE can improve group performance depends on intra-group psychological processes, which depend on the group members' perceptions and expectations about PCE. Imagine a group where some members take a certain PCE and others do not. If the non-using group members overestimate the efficacy of this PCE, they might rely more on the performance of the users in the group and themselves exert less effort to contribute to the group's goal. By causing such “social loafing” (Latané et al., 1979) the PCE could even reduce the performance of the group. Hence, a PCE technique that is an enhancement of individual performance for pharmacological reasons can act as an impairment for a group for psychological reasons like a misperception of efficacy (cf. Faber et al., 2015b).

## Conclusion

We conclude that both currently available PCE and NPCE techniques can enhance human cognition to a significant, albeit moderate degree and that both are subject to moderating variables. While the actual effectiveness of both types of enhancement appears to be similar, their public perception, which in large part follows perceptions of effectiveness, is not. We hope that future research will attempt to quantitatively compare the effectiveness of PCE and NPCE, which may lead to a more balanced debate about the possibilities of cognitive enhancement.

### Conflict of interest statement

The authors declare that the research was conducted in the absence of any commercial or financial relationships that could be construed as a potential conflict of interest.

## Footnotes

^1^Note that all effect sizes refer to Cohen's d (or its adjusted versions like Hedge's g), whereby small effect sizes are defined by a value around 0.2, moderate effect sizes by around 0.5, and large effect sizes by around 0.8 (Cohen, 1992). Where available, we report the exact effect sizes.
